# Adverse events analysis of Relugolix (Orgovyx^®^) for prostate cancer based on the FDA Adverse Event Reporting System (FAERS)

**DOI:** 10.1371/journal.pone.0312481

**Published:** 2024-10-22

**Authors:** Ruibo Li, Xi Chen, Yujie Wang

**Affiliations:** 1 Department of Orthopaedics, Deyang Peoples’ Hospital, Deyang, Sichuan Province, China; 2 Department of Urology, Zigong Fourth People’s Hospital, Zigong City, Sichuan Province, China; Teikyo University, School of Medicine, JAPAN

## Abstract

**Background:**

Due to the limitations of clinical trials, some delayed and rare adverse events (AEs) may remain undetected, and safety information can be supplemented through post-market data analysis. This study aims to comprehensively analyze the AEs associated with Relugolix (Orgovyx^®^) using data from the FAERS database, and gain a better understanding of the potential risks and side effects of Relugolix (Orgovyx^®^) therapy.

**Methods:**

Data of Relugolix (Orgovyx^®^) were collected from the FAERS database covering the period from the fourth quarter of 2020 to the third quarter of 2023. Disproportionality analysis was performed by calculating the reporting odds ratios (ROR), proportional reporting ratio (PRR), Bayesian analysis confidence propagation neural network (BCPNN), and the multi-item gamma Poisson shrinker (MGPS) to detect positive signals.

**Results:**

Totally, 5,382,189 reports were collected from the FAERS database, 4,397 reports of Relugolix (Orgovyx^®^) were identified as the ‘primary suspected (PS)’ AEs. Relugolix (Orgovyx^®^) induced AEs occurred in 26 organ systems. 58 significant disproportionality preferred terms (PTs) satisfying with the four algorithms were retained at the same time. Unexpected significant AEs such as Pollakiuria, and Prostatic specific antigen increased also occur. The median time of onset was 60 days. The majority of the AEs occurred within the first 30 days after Relugolix (Orgovyx^®^) initiation.

**Conclusion:**

Common AEs included Hot flush, Fatigue, Asthenia, Constipation, and Myalgia. These AEs should be focused on when using the drug to avoid serious consequences. In addition, the study results also suggested that the drug may exist Pollakiuria, Prostatic specific antigen increased and other AEs not mentioned in the manual, to supplement the AEs in the manual. This study is helpful for clinicians and pharmacists to improve their understanding of Relugolix (Orgovyx^®^) related AEs, and take timely prevention and treatment measures to ensure drug safety for patients.

## Introduction

Prostate cancer is a prevalent and significant health concern among aging men. Androgen deprivation therapy (ADT), which involves suppressing testosterone production, is one of the main treatments for advanced prostate cancer [1].

Recently, a notable addition to the ADT arsenal has been Relugolix (Orgovyx^®^), an oral gonadotropin-releasing hormone (GnRH) antagonist. Its approval by the US Food and Drug Administration (FDA) was based on the compelling efficacy and safety data from the HERO trial [2, 3]. As the sole orally-administered GnRH receptor antagonist on the market, Relugolix offers distinct advantages over its injectable counterparts. Specifically, it circumvents the potential adverse effects linked to GnRH agonists, such as tumor flare, and obviates the need for frequent injections, thereby eliminating injection site reactions—a common issue with injectable GnRH antagonists like Degarelix [2, 4, 5].

Moreover, Relugolix exhibits a rapid onset of action, swiftly suppressing testosterone levels within hours of administration and maintaining this suppression consistently throughout the dosing interval. This steady suppression minimizes the risk of testosterone escape, a prevalent concern with LHRH agonists, potentially leading to improved outcomes for patients [2, 4].

Due to the limitations of clinical trials, some delayed and rare adverse events (AEs) may remain undetected, and safety information can be supplemented through post-market data analysis. In recent years, the FDA Adverse Event Reporting System (FAERS) has become an invaluable resource for assessing the safety profile of various pharmaceutical interventions. The FAERS database serves a repository of reports filed by healthcare professionals, patients, and manufacturers, enabling the monitoring and analysis of AEs associated with various medications. Understanding the safety profile of Relugolix (Orgovyx^®^) is crucial as it allows healthcare providers to make informed decisions and optimize patient care.

Therefore, this study aims to perform an AEs analysis of Relugolix (Orgovyx^®^) based on the FAERS. By systematically examining reported AEs, we seek to identify patterns, evaluate the severity and frequency of these events, and provide insights into the safety and tolerability of Relugolix (Orgovyx^®^) as an innovative treatment option for prostate cancer.

Through this analysis, we anticipate contributing valuable information to healthcare professionals, regulatory agencies, and patients regarding the potential risks and benefits associated with Relugolix (Orgovyx^®^). Such insights will facilitate evidence-based decision-making, support the development of guidelines for optimal drug usage, and further enhance patient safety in the management of prostate cancer.

## Materials and methods

### Data source and collection

The data in this study were derived from the FAERS database in the United States. The FAERS database, which has been freely available since 2004, collects post-marketing AEs and is updated quarterly. AEs were collected from the fourth quarter of 2020 to the third quarter of 2023 based on the launch date of Relugolix (Orgovyx^®^). All data used in this study were obtained from publicly available databases; further ethical approval was not required.

### Data processing

**Fig 1:**
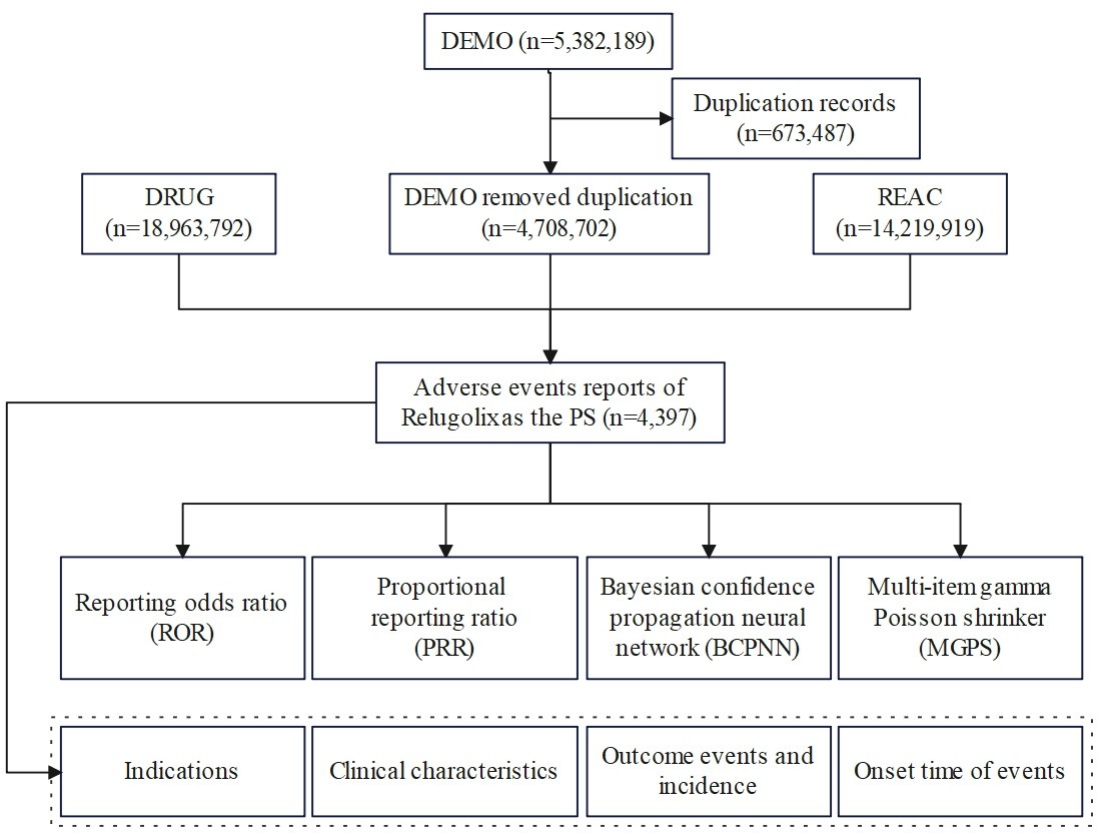
Flow diagram of the study. DEMO, demographic and administrative information; DRUG, drug Information; REAC, preferred terminology for adverse drug reactions; PS, primary suspect drug.

Write the downloaded XML data package into RStudio and clean the data following the recommendations from the FDA. Perform a query using the generic name " Relugolix" and the trade name " Orgovyx" as targe drugs, and include only AEs reports where Relugolix (Orgovyx^®^) is the primary suspected drug (PS). Identify and remove duplicate reports based on the report information. For AEs names in the reports, use the preferred term (PT) from the Medical Dictionary for Regulatory Activities (MedDRA) for standardized encoding. Clinical characteristics including gender, age, reporting country, reporter, reporting time and outcomes of patients with Relugolix (Orgovyx^®^) -related AEs were collected. All AEs reports for Relugolix (Orgovyx^®^) were analyzed at the System Organ Class (SOC) and PT levels. Additionally, we assessed the time-to-onset of AEs caused by Relugolix (Orgovyx^®^) [6]. The flow diagram of our study is shown in Fig 1.

### Statistical analysis

Descriptive analysis was used to show the characteristics of all AEs reports regarding to Relugolix (Orgovyx^®^). Disproportionality analysis, which is widely used in pharmacovigilance study, was performed to identify potential signals between Relugolix (Orgovyx^®^) and all AEs in our investigation. Reporting odds ratio (ROR), the proportional reporting ratio (PRR), the Bayesian confidence propagation neural network (BCPNN), and the multi-item gamma Poisson shrinker (MGPS) are four major specific indices that were calculated using standard formulas to assess potential associations between Relugolix (Orgovyx^®^) and AEs [6, 7]. The equations and criteria for the four algorithms are described in Table 1. AEs signals that satisfied all four algorithm criteria were considered significant signals. Significant signals not listed in the package insert were considered new signals. Additionally, the onset time was defined as the date of initiation of drug use to the time of occurrence of adverse reactions [8]. Descriptive analysis and disequilibrium analysis provided sufficient information to describe Relugolix (Orgovyx^®^) AEs in the treatment of prostate cancer, including the distribution and trends of AEs, so further regression analysis was not necessary. Data processing was carried out using Microsoft Excel 2023 and RStudio (Version 4.3.1.).

**Table 1:** Four major algorithms used for signal detection.

Algorithms	Equation	Criteria
ROR	ROR = ad/b/c	lower limit of 95% CI>1, N≥3
	95%CI = e^ln(ROR)±1.96(1/a+1/b+1/c+1/d)^0.5^	
PRR	PRR = a(c+d)/c/(a+b)	PRR≥2, χ^2^≥4, N≥3
	χ^2^ = [(ad-bc)^2](a+b+c+d)/[(a+b)(c+d)(a+c)(b+d)]	
BCPNN	$\text{IC}={\text{log}}_{2}\frac{\text{a}\left(\text{a}+\text{b}+\text{c}+\text{d}\right)}{\left(\text{a}+\text{c}\right)\left(\text{a}+\text{b}\right)}$	IC025>0
	95%CI = E(IC) ± 2V(IC)^0.5	
MGPS	$\text{EBGM}=\frac{a(a+b+c+d)}{\left(a+c\right)\left(a+b\right)}$	EBGM05>2
	95%CI = e^ln(EBGM)±1.96(1/a+1/b+1/c+1/d)^0.5^	

Algorithms: ROR, reporting odds ratio; PRR, proportional reporting ratio; BCPNN, Bayesian confidence propagation neural network; MGPS, multi-item gamma Poisson shrinker.

Equation: a, number of reports containing both the target drug and target adverse drug reaction; b, number of reports containing other adverse drug reaction of the target drug; c, number of reports containing the target adverse drug reaction of other drugs; d, number of reports containing other drugs and other adverse drug reactions. 95%CI, 95% confidence interval; *N*, the number of reports; χ^2^, chi-squared; IC, information component; IC025, the lower limit of 95% CI of the IC; E(IC), the IC expectations; V(IC), the variance of IC; EBGM, empirical Bayesian geometric mean; EBGM05, the lower limit of 95% CI of EBGM.

## Results

### General characteristics

This study carried out a comprehensive analysis of 5,382,189 AEs in the FAERS database, of which 4,397 reports were primarily associated with Relugolix (Orgovyx^®^). Among these Relugolix (Orgovyx^®^)-related AEs reports, men accounted for about 95.34%. Regarding age distribution, patients aged 65~85 reported AEs most frequently, accounting for 28.59%. The primary reporters of these AEs were consumers (83.83%) and health professionals (10.39%). From the level of reporting countries, the United States reported the highest proportion, up to 96.82%, followed by Japan, the proportion of 2.02%. Time series analysis showed that 387 cases were reported in 2021, 2,098 cases in 2022, and 1,912 cases in the first three quarters of 2023, showing an overall upward trend. Among the clinical outcomes of AEs, other important AEs accounted for about 7.12%, followed by hospitalization or extended hospitalization (5.39%), and death related reports (4.75%) (Table 2).

**Table 2:** Clinical characteristics of adverse events to Relugolix (Orgovyx^®^).

Characteristics	Case number, n	Case proportion, %
**Number of cases**	4397	
**Gender**		
Female	142	3.23
Male	4192	95.34
Unknown	63	1.43
**Age (year)**		
<18	1	0.02
18~64	171	3.89
65~85	336	7.64
>85	1257	28.59
Unknown	2632	59.86
**Reported Person**		
Consumer	3686	83.83
Health Professional	457	10.39
Physician	203	4.62
Pharmacist	39	0.89
Unknown	12	0.27
**Reported Countries (Top five)**		
United State	4257	96.82
Japan	89	2.02
Germany	17	0.39
Portugal	7	0.16
Sweden	6	0.14
**Reporting year**		
2021	387	8.80
2022	2098	47.71
2023	1912	43.48
**Serious outcome**		
Other Serious (Important Medical Event)	313	7.12
Hospitalization (initial or prolonged)	237	5.39
Death	209	4.75
Disability	5	0.11
Life-Threatening	5	0.11
Required Intervention to Prevent Permanent	1	0.02
Unknown	3627	82.49

### Signal detection

The signal strength of Relugolix (Orgovyx^®^) at the SOC level is reported in Table 3. Statistically, we found that Relugolix (Orgovyx^®^)-induced AEs involved 26 organ systems. The most commonly reported SOCs are Surgical and medical procedures, General disorders and administration site conditions, and Vascular disorders. The significant SOCs that met the four criteria were Surgical and medical procedures and Vascular disorders.

**Table 3:** The signal strength of Relugolix (Orgovyx^®^) at the System Organ Class (SOC) level.

System organ class (SOC)	Relugolix (Orgovyx^®^) cases reporting SOC	ROR (95% two-sided CI)	PRR (χ^2^)	EBGM(EBGM05)	IC (IC025)
Surgical and medical procedures	1648	15.27 (14.48–16.11)	12.7 (17874)	12.60 (11.95)	3.66 (1.99)
General disorders and administration site conditions	1322	0.78 (0.74–0.83)	0.81 (66.3)	0.82 (0.77)	-0.29 (-1.96)
Vascular disorders	802	5.17 (4.81–5.56)	4.81 (2455.7)	4.80 (4.46)	2.26 (0.60)
Investigations	685	1.29 (1.19–1.39)	1.27 (41.4)	1.27 (1.17)	0.34 (-1.32)
Gastrointestinal disorders	644	0.91 (0.84–0.98)	0.92 (5.29)	0.92 (0.85)	-0.13 (-1.79)
Musculoskeletal and connective tissue disorders	638	1.38 (1.27–1.49)	1.35 (61.0)	1.35 (1.25)	0.43 (-1.23)
Nervous system disorders	615	0.94 (0.86–1.02)	0.95 (2.02)	0.95 (0.87)	-0.08 (-1.75)
Injury, poisoning and procedural complications	611	0.51 (0.46–0.55)	0.54 (275.3)	0.54 (0.50)	-0.89 (-2.56)
Psychiatric disorders	360	0.69 (0.62–0.76)	0.70 (48.98)	0.70 (0.63)	-0.51 (-2.18)
Skin and subcutaneous tissue disorders	337	0.72 (0.65–0.81)	0.74 (33.94)	0.73 (0.66)	-0.44 (-2.11)
Renal and urinary disorders	219	1.31 (1.14–1.49)	1.30 (15.36)	1.30 (1.14)	0.38 (-1.29)
Infections and infestations	202	0.38 (0.33–0.44)	0.4 (199)	0.39 (0.34)	-1.34 (-3.01)
Product issues	177	1.05 (0.91–1.22)	1.05 (0.43)	1.05 (0.90)	0.07 (-1.60)
Respiratory, thoracic and mediastinal disorders	157	0.38 (0.32–0.45)	0.39 (155.5)	0.39 (0.33)	-1.35 (-3.02)
Reproductive system and breast disorders	155	2.95 (2.51–3.45)	2.91 (195.4)	2.91 (2.48)	1.54 (-0.12)
Metabolism and nutrition disorders	139	0.82 (0.69–0.97)	0.82 (5.48)	0.82 (0.69)	-0.28 (-1.95)
Neoplasms benign, malignant and unspecified (incl cysts and polyps)	121	0.26 (0.22–0.31)	0.27 (248.5)	0.27 (0.23)	-1.88 (-3.55)
Cardiac disorders	121	0.69 (0.57–0.82)	0.69 (17.16)	0.69 (0.58)	-0.53 (-2.2)
Eye disorders	48	0.27 (0.2–0.36)	0.28 (93.5)	0.28 (0.21)	-1.86 (-3.53)
Blood and lymphatic system disorders	35	0.22 (0.16–0.31)	0.23 (94.3)	0.23 (0.16)	-2.14 (-3.81)
Ear and labyrinth disorders	27	0.74 (0.51–1.08)	0.74 (2.48)	0.74 (0.51)	-0.43 (-2.1)
Immune system disorders	27	0.27 (0.2–0.39)	0.27 (52.82)	0.27 (0.19)	-1.87 (-3.54)
Social circumstances	26	0.61 (0.41–0.9)	0.61 (6.61)	0.61 (0.41)	-0.72 (-2.38)
Hepatobiliary disorders	14	0.19 (0.12–0.33)	0.2 (46.93)	0.19 (0.12)	-2.36 (-4.02)
Pregnancy, puerperium and perinatal conditions	6	0.2 (0.1–0.45)	0.2 (18.7)	0.20 (0.09)	-2.29 (-3.96)
Endocrine disorders	2	0.08 (0.02–0.34)	0.08 (19.8)	0.08 (0.02)	-3.56 (-5.22)

ROR, reporting odds ratio; CI, confidence interval; PRR, proportional reporting ratio; χ^2^, chi-squared; EBGM, empirical Bayesian geometric mean; EBGM05, the lower limit of 95% CI of EBGM; IC, information component; IC 025, the lower limit of 95% CI of the IC.

After excluding signals unrelated to drug treatment, such as product issues, various injuries, poisonings, procedure-related complications, surgeries, and medical operations, a total of 58 PTs were identified as significant signals that met the criteria of all four algorithms (Table 4). The top five PTs reported most frequently were Hot flush, Fatigue, Asthenia, Constipation, and Myalgia. In addition, these five AEs are also recorded in the drug instructions [9]. The top five PTs in terms of correlation strength are Uterine myoma expulsion Male genital atrophy, Blood testosterone increased, Hot flush, and Blood testosterone abnormal.

**Table 4:** Signal strength of reports of Relugolix (Orgovyx^®^) at the Preferred Term (PT) level.

System organ class (SOC)	Preferred terms (PTs)	Relugolix (Orgovyx^®^) cases reporting PT	ROR (95% two-sided CI)	PRR (χ^2^)	EBGM(EBGM05)	IC (IC025)
Vascular disorders	Hot flush	716	79.33 (73.39–85.77)	73.20 (48749.49)	69.95 (65.54)	6.13 (4.46)
Cardiac disorders	Coronary artery occlusion	5	4.84 (2.01–11.65)	4.839 (15.18)	4.83 (2.31)	2.27 (0.60)
Gastrointestinal disorders	Constipation	126	4.10 (3.44–4.89)	4.06 (290.73)	4.05 (3.50)	2.02 (0.35)
	Faeces hard	5	8.16 (3.39–19.66)	8.16 (31.25)	8.12 (3.89)	3.02 (1.35)
General disorders and administration site conditions	Fatigue	465	4.07 (3.70–4.46)	3.91 (1017.67)	3.90 (3.61)	1.96 (0.30)
	Asthenia	177	3.69 (3.18–4.28)	3.63 (338.85)	3.62 (3.20)	1.86 (0.20)
	Feeling cold	18	5.03 (3.16–7.99)	5.02 (57.85)	5.01 (3.40)	2.33 (0.66)
	Fat tissue increased	7	37.44 (17.68–79.25)	37.41 (242.22)	36.55 (19.52)	5.19 (3.52)
	Exercise tolerance decreased	5	5.65 (2.35–13.61)	5.65 (19.07)	5.63 (2.70)	2.49 (0.83)
Investigations	Blood glucose increased	77	3.74 (2.99–4.69)	3.72 (153.10)	3.71 (3.08)	1.90 (0.23)
	Prostatic specific antigen increased	58	30.90 (23.81–40.10)	30.71 (1635.17)	30.14 (24.23)	4.91 (3.25)
	Blood testosterone increased	18	81.29 (50.59–130.63)	81.14 (1354.08)	77.16 (51.89)	6.27 (4.60)
	Glycosylated haemoglobin increased	17	4.79 (2.97–7.72)	4.79 (50.85)	4.78 (3.21)	2.26 (0.59)
	Blood testosterone abnormal	13	72.82 (41.74–127.05)	72.72 (878.50)	69.52 (43.64)	6.12 (4.44)
	Prostatic specific antigen abnormal	13	59.72 (34.31–103.96)	59.64 (721.83)	57.47 (36.14)	5.85 (4.17)
	Liver function test increased	13	3.59 (2.08–6.19)	3.58 (24.20)	3.58 (2.27)	1.84 (0.17)
	Blood triglycerides increased	12	8.44 (4.78–14.89)	8.43 (78.20)	8.39 (5.22)	3.07 (1.40)
	Blood urine present	10	4.50 (2.42–8.38)	4.4985 (27.14)	4.49 (2.67)	2.17 (0.50)
	Blood thyroid stimulating hormone increased	7	7.18 (3.42–15.09)	7.176 (37.04)	7.15 (3.84)	2.84 (1.17)
	Electrocardiogram abnormal	5	4.85 (2.02–11.67)	4.848 (15.22)	4.84 (2.32)	2.27 (0.61)
	Renal function test abnormal	4	5.53 (2.07–14.75)	5.524 (14.77)	5.51 (2.42)	2.46 (0.79)
Metabolism and nutrition disorders	Increased appetite	10	4.85 (2.60–9.03)	4.8461 (30.44)	4.83 (2.87)	2.27 (0.61)
	Diabetes mellitus inadequate control	9	4.04 (2.10–7.78)	4.0393 (20.53)	4.03 (2.33)	2.01 (0.34)
	Weight loss poor	5	10.93 (4.53–26.34)	10.92 (44.75)	10.85 (5.20)	3.44 (1.77)
	Glucose tolerance impaired	4	5.08 (1.90–13.57)	5.082 (13.07)	5.07 (2.23)	2.34 (0.67)
Musculoskeletal and connective tissue disorders	Myalgia	96	5.10 (4.17–6.23)	5.05 (311.82)	5.04 (4.26)	2.33 (0.67)
	Muscle atrophy	29	20.92 (14.50–30.20)	20.86 (541.15)	20.60 (15.15)	4.36 (2.70)
	Bone pain	27	3.74 (2.56–5.46)	3.73 (53.98)	3.73 (2.72)	1.90 (0.23)
Neoplasms benign, malignant and unspecified (incl cysts and polyps)	Metastases to bone	15	5.64 (3.39–9.37)	5.63 (56.99)	5.62 (3.67)	2.50 (0.82)
	Prostate cancer metastatic	9	17.45 (9.04–33.66)	17.43 (137.85)	17.25 (9.95)	4.11 (2.44)
	Metastases to spine	7	14.49 (6.88–30.50)	14.476 (87.01)	14.35 (7.70)	3.84 (2.17)
	Metastases to lymph nodes	7	7.18 (3.41–15.08)	7.171 (37.01)	7.14 (3.84)	2.84 (1.17)
	Uterine myoma expulsion	6	4668.46 (942.11–23133.76)	4665.4 (6995.1)	1167.10 (305.85)	10.19 (8.26)
Nervous system disorders	Lethargy	24	3.91 (2.62–5.84)	3.90 (51.79)	3.90 (2.79)	1.96 (0.30)
	Dizziness postural	13	9.05 (5.24–15.61)	9.04 (92.40)	8.99 (5.69)	3.17 (1.50)
	Presyncope	10	3.47 (1.86–6.45)	3.4666 (17.52)	3.46 (2.06)	1.79 (0.12)
	Hypotonia	6	6.19 (2.77–13.80)	6.183 (25.97)	6.16 (3.15)	2.62 (0.96)
Psychiatric disorders	Libido decreased	26	22.13 (15.02–32.61)	22.07 (515.76)	21.78 (15.74)	4.45 (2.78)
	Mood swings	21	7.18 (4.67–11.02)	7.16 (110.89)	7.14 (4.98)	2.83 (1.17)
	Bradyphrenia	16	17.40 (10.63–28.50)	17.38 (244.23)	17.20 (11.38)	4.10 (2.44)
	Loss of libido	11	11.08 (6.12–20.06)	11.07 (100.09)	11.00 (6.70)	3.46 (1.79)
Renal and urinary disorders	Pollakiuria	68	13.23 (10.41–16.81)	13.14 (756.61)	13.04 (10.67)	3.70 (2.04)
	Dysuria	28	6.22 (4.29–9.01)	6.20 (121.67)	6.18 (4.53)	2.63 (0.96)
	Nocturia	26	15.14 (10.29–22.30)	15.10 (339.20)	14.97 (10.83)	3.90 (2.24)
	Micturition urgency	20	13.86 (8.92–21.54)	13.84 (236.10)	13.72 (9.49)	3.78 (2.11)
	Urinary incontinence	12	3.42 (1.94–6.03)	3.42 (20.52)	3.42 (2.13)	1.77 (0.10)
	Micturition disorder	5	11.02 (4.57–26.56)	11.01 (45.20)	10.94 (5.24)	3.45 (1.78)
Reproductive system and breast disorders	Erectile dysfunction	21	6.78 (4.41–10.41)	6.77 (102.80)	6.74 (4.71)	2.75 (1.09)
	Gynaecomastia	18	6.31 (3.97–10.03)	6.29 (79.91)	6.28 (4.26)	2.65 (0.98)
	Intermenstrual bleeding	15	10.23 (6.15–17.01)	10.21 (123.87)	10.15 (6.64)	3.35 (1.68)
	Genital haemorrhage	12	10.28 (5.82–18.15)	10.27 (99.77)	10.21 (6.35)	3.35 (1.68)
	Testicular pain	7	19.81 (9.40–41.76)	19.79 (123.34)	19.56 (10.48)	4.29 (2.62)
	Sexual dysfunction	6	4.27 (1.92–9.53)	4.272 (15.00)	4.26 (2.18)	2.09 (0.42)
	Male genital atrophy	5	388.99 (145.96–1036.68)	388.78 (1547.14)	311.23 (137.05)	8.28 (6.52)
	Testicular atrophy	5	53.29 (21.85–129.97)	53.26 (247.90)	51.53 (24.44)	5.69 (4.00)
	Uterine haemorrhage	5	10.21 (4.24–24.60)	10.20 (41.24)	10.14 (4.86)	3.34 (1.67)
Skin and subcutaneous tissue disorders	Hyperhidrosis	63	4.08 (3.19–5.23)	4.06 (145.35)	4.05 (3.29)	2.02 (0.35)
	Night sweats	54	12.99 (9.93–17.00)	12.92 (589.32)	12.82 (10.24)	3.68 (2.01)

ROR, reporting odds ratio; CI, confidence interval; PRR, proportional reporting ratio; χ^2^, chi-squared; EBGM, empirical Bayesian geometric mean; EBGM05, the lower limit of 95% CI of EBGM; IC, information component; IC 025, the lower limit of 95% CI of the IC.

Interestingly, 32 of the 58 significant PTs were not recorded in the drug’s instruction manual (Table 5). Among them, according to the frequency of reporting, the top 10 are Pollakiuria, Prostatic specific antigen increased, Muscle atrophy, Dysuria, Nocturia, Erectile dysfunction, Micturition urgency, Blood testosterone increased, Gynaecomastia, and Feeling cold.

**Table 5:** Signal strength of adverse events that are unexpected findings of Relugolix (Orgovyx^®^)-related adverse events at the preferred term (PT) level.

Preferred terms (PTs)	Relugolix (Orgovyx^®^) cases reporting PT	ROR (95% two-sided CI)	PRR (χ^2^)	EBGM(EBGM05)	IC (IC025)
Pollakiuria	68	13.23 (10.41–16.81)	13.14 (756.61)	13.04 (10.67)	3.70 (2.04)
Prostatic specific antigen increased	58	30.90 (23.81–40.10)	30.71 (1635.17)	30.14 (24.23)	4.91 (3.25)
Muscle atrophy	29	20.92 (14.50–30.20)	20.86 (541.15)	20.60 (15.15)	4.36 (2.70)
Dysuria	28	6.22 (4.29–9.01)	6.20 (121.67)	6.18 (4.53)	2.63 (0.96)
Nocturia	26	15.14 (10.29–22.30)	15.10 (339.20)	14.97 (10.83)	3.90 (2.24)
Erectile dysfunction	21	6.78 (4.41–10.41)	6.77 (102.80)	6.74 (4.71)	2.75 (1.09)
Micturition urgency	20	13.86 (8.92–21.54)	13.84 (236.10)	13.72 (9.49)	3.78 (2.11)
Feeling cold	18	5.03 (3.16–7.99)	5.02 (57.85)	5.01 (3.40)	2.33 (0.66)
Blood testosterone increased	18	81.29 (50.59–130.63)	81.14 (1354.08)	77.16 (51.89)	6.27 (4.60)
Gynaecomastia	18	6.31 (3.97–10.03)	6.29 (79.91)	6.28 (4.26)	2.65 (0.98)
Glycosylated haemoglobin increased	17	4.79 (2.97–7.72)	4.79 (50.85)	4.78 (3.21)	2.26 (0.59)
Bradyphrenia	16	17.40 (10.63–28.50)	17.38 (244.23)	17.20 (11.38)	4.10 (2.44)
Blood testosterone abnormal	13	72.82 (41.74–127.05)	72.72 (878.50)	69.52 (43.64)	6.12 (4.44)
Prostatic specific antigen abnormal	13	59.72 (34.31–103.96)	59.64 (721.83)	57.47 (36.14)	5.85 (4.17)
Dizziness postural	13	9.05 (5.24–15.61)	9.04 (92.40)	8.99 (5.69)	3.17 (1.50)
Urinary incontinence	12	3.42 (1.94–6.03)	3.42 (20.52)	3.42 (2.13)	1.77 (0.10)
Increased appetite	10	4.85 (2.60–9.03)	4.8461 (30.44)	4.83 (2.87)	2.27 (0.61)
Presyncope	10	3.47 (1.86–6.45)	3.4666 (17.52)	3.46 (2.06)	1.79 (0.12)
Prostate cancer metastatic	9	17.45 (9.04–33.66)	17.43 (137.85)	17.25 (9.95)	4.11 (2.44)
Fat tissue increased	7	37.44 (17.68–79.25)	37.41 (242.22)	36.55 (19.52)	5.19 (3.52)
Blood thyroid stimulating hormone increased	7	7.18 (3.42–15.09)	7.176 (37.04)	7.15 (3.84)	2.84 (1.17)
Metastases to spine	7	14.49 (6.88–30.50)	14.476 (87.01)	14.35 (7.70)	3.84 (2.17)
Metastases to lymph nodes	7	7.18 (3.41–15.08)	7.171 (37.01)	7.14 (3.84)	2.84 (1.17)
Testicular pain	7	19.81 (9.40–41.76)	19.79 (123.34)	19.56 (10.48)	4.29 (2.62)
Uterine myoma expulsion	6	4668.46 (942.11–23133.76)	4665.4 (6995.1)	1167.10 (305.85)	10.19 (8.26)
Hypotonia	6	6.19 (2.77–13.80)	6.183 (25.97)	6.16 (3.15)	2.62 (0.96)
Sexual dysfunction	6	4.27 (1.92–9.53)	4.272 (15.00)	4.26 (2.18)	2.09 (0.42)
Faeces hard	5	8.16 (3.39–19.66)	8.16 (31.25)	8.12 (3.89)	3.02 (1.35)
Exercise tolerance decreased	5	5.65 (2.35–13.61)	5.65 (19.07)	5.63 (2.70)	2.49 (0.83)
Micturition disorder	5	11.02 (4.57–26.56)	11.01 (45.20)	10.94 (5.24)	3.45 (1.78)
Male genital atrophy	5	388.99 (145.96–1036.68)	388.78 (1547.14)	311.23 (137.05)	8.28 (6.52)
Testicular atrophy	5	53.29 (21.85–129.97)	53.26 (247.90)	51.53 (24.44)	5.69 (4.00)

ROR, reporting odds ratio; CI, confidence interval; PRR, proportional reporting ratio; χ^2^, chi-squared; EBGM, empirical Bayesian geometric mean; EBGM05, the lower limit of 95% CI of EBGM; IC, information component; IC 025, the lower limit of 95% CI of the IC.

### Onset time of events

**Fig 2:**
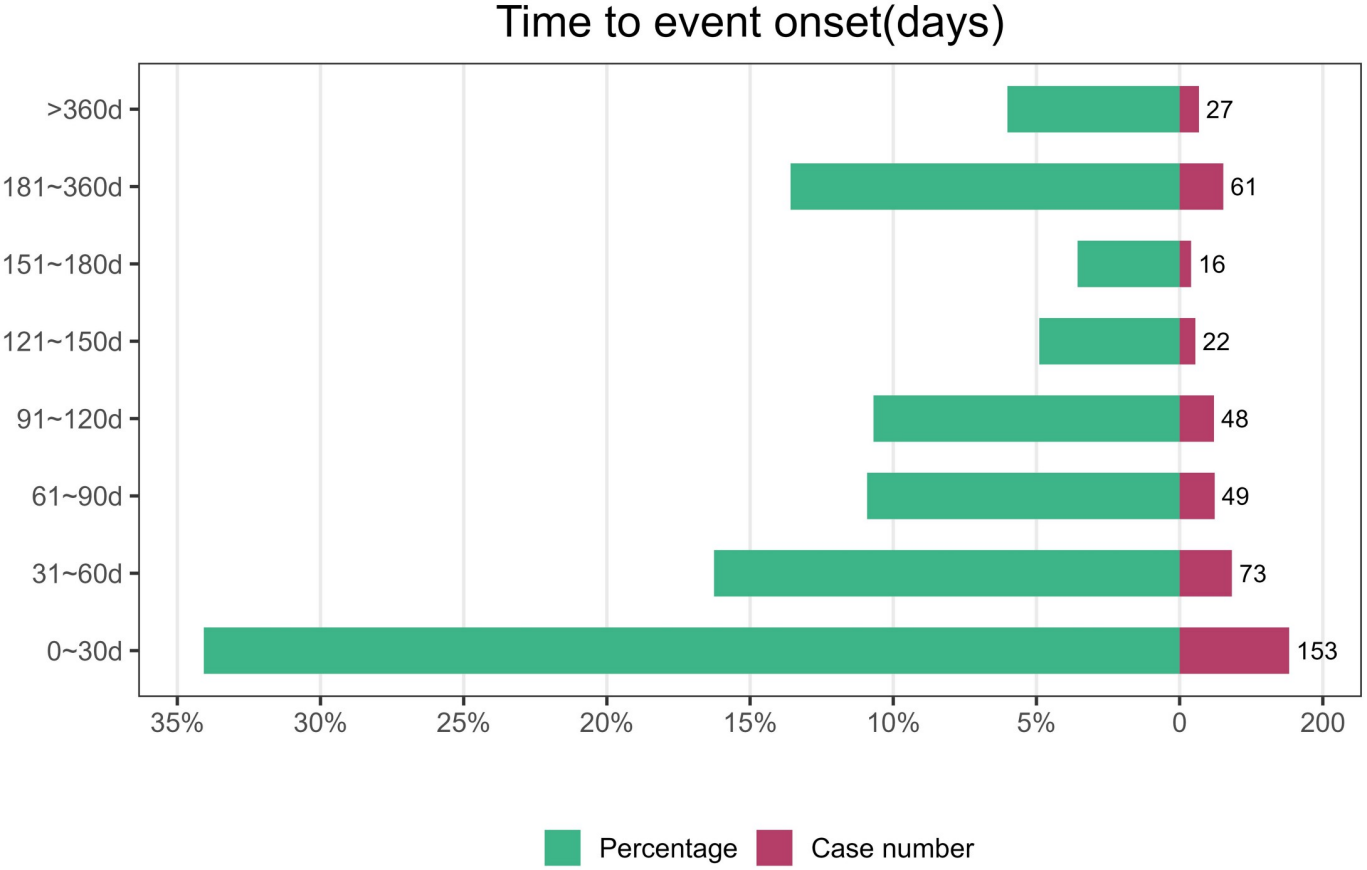
Time to onset of Relugolix (Orgovyx^®^)-related adverse events.

Excluding false positives, a total of 449 cases reported onset time, with a median onset time of 60 days. Most AEs occurred within 30 days of medication, accounting for 34.08%. The AEs showed a decreasing trend after more than 30 days of treatment (Fig 2).

## Discussion

Relugolix (Orgovyx^®^) is used primarily for the treatment of advanced prostate cancer in men. Interestingly, in the results of this study, we found that 3.23% of women reported AEs. In addition, among the significant PTs screened after disproportionation analysis, some PTs appeared only in female patients, such as Uterine myoma expulsion, Intermenstrual bleeding and Uterine haemorrhage. The reason why this phenomenon occurs is that in clinical practice, there are cases of off-label use of Relugolix (Orgovyx^®^). Several high-quality studies have demonstrated that Relugolix (Orgovyx^®^) can not only effectively reduce uterine bleeding and pain symptoms in patients with uterine fibroids, but also significantly improve endometriosis related pain and is well tolerated [10–12]. An oral fixeddose combination (FDC) of Relugolix/estradiol/norethisterone (also known as norethindrone) acetate 40/1/0.5 mg [Ryeqo^®^ (EU); Myfembree^®^ (USA)] has been approved for use in women with symptomatic uterine fibroids [13, 14]. In addition, it is approved in the USA as a treatment for endometrium-related pain [15].

At the SOC level, besides Surgical and medical procedures, the highest reported frequency is General disorders and administration site conditions. Within the General disorders and administration site conditions category, the most reported AEs is Fatigue, followed by Asthenia. Both of these AEs are documented in the drug’s prescribing information and are mostly grade 1 or 2, consistent with the physiological effects of testosterone suppression [2].

The SOC with the highest variety of PT signals is Investigations, followed by Reproductive system and breast disorders, and Neoplasms benign, malignant and unspecified (including cysts and polyps). In Investigations, the most common AEs are Blood glucose increased and Prostatic specific antigen increased, which align with the drug’s prescribing information and previous HERO trial results.^2^ These events are attributed to metabolic abnormalities caused by testosterone suppression and changes in body composition, including Metabolism and nutrition disorders such as Weight loss poor and Glucose tolerance impaired.

At the PT level, the top five reported PTs in this study are all documented in the drug’s prescribing information, further highlighting the reliability of this study. The most frequent PT is Hot flush, classified under Vascular disorders. Additionally, there are relatively severe AEs related to the cardiovascular system, such as Coronary artery occlusion. This study did not find other common cardiovascular AEs seen in androgen deprivation therapy, such as hypertension and stroke. One of the significant advantages of Relugolix (Orgovyx^®^) compared to GnRH agonists and injectable GnRH agonist Leuprolide is the significant reduction in the incidence of cardiovascular events [2, 4, 5]. The specific mechanisms behind cardiovascular AEs during androgen deprivation therapy are still unclear. Active management of cardiovascular risk factors and primary prevention with aspirin and/or statins form the basis of treating patients at increased risk of arterial events. In such cases, new medications like low-dose anticoagulants have also shown clinical benefits [16, 17]. Although the risk of cardiovascular disease increases in prostate cancer patients, specific clinical guidelines for reducing cardiovascular disease risk have not been established [18]. Further research is needed to determine the best strategies for reducing cardiovascular events in prostate cancer patients receiving hormone therapy [17].

In this study, a total of 32 unexpected AEs not recorded in the drug instruction manual were identified. Among top 10 frequency PT signals, Pollakiuria and Prostatic specific antigen increased were not listed in the drug instruction manual. Among top 10 signal strengths PT signals, Male genital atrophy, Blood testosterone increased, Testicular atrophy, Fat tissue increased, and Libido decreased were also not listed, indicating novel potential risk signals. However, there were limited case reports and continuous monitoring is warranted to avoid serious consequences.

Our study revealed a median onset time of 60 days, with the majority of cases (n = 153, 34.08%) occurring within the first month of treatment with Relugolix (Orgovyx^®^). These results suggest that close attention should be paid to AEs occurring within the first month of treatment with Relugolix (Orgovyx^®^) to enable early detection and minimize potential life-threatening complications for patients.

There are several limitations to this study. Firstly, some reports in the FAERS database originate from patients’ spontaneous reports, which may vary in quality, and only recorded observed AEs, inevitably leading to underreporting or reporting bias. Secondly, the disproportionality analysis only provides an estimate of signal strength, which is statistically significant but cannot offer direct correlation. Thirdly, there are multiple unmeasured confounding factors, such as potential drug-drug interactions, comorbidities, and reported chain reactions, that may affect the actual recording of AEs [19], making it difficult to determine the true incidence of AEs from FAERS data.

## Conclusion

This study comprehensively analyzed post-marketing AEs of Relugolix (Orgovyx^®^) through the FAERS database, and found that common AEs included Hot flush, Fatigue, Asthenia, Constipation, and Myalgia. These AEs should be focused on when using the drug to avoid serious consequences. In addition, the study results also suggested that the drug may exist Pollakiuria, Prostatic specific antigen increased and other AEs not mentioned in the manual, to supplement the AEs in the manual. This study is helpful for clinicians and pharmacists to improve their understanding of Relugolix (Orgovyx^®^) related AEs, and take timely prevention and treatment measures to ensure drug safety for patients.

## Abbreviations

ADT: androgen deprivation therapy
AEs: adverse events
BCPNN: Bayesian analysis confidence propagation neural network
DEMO: demographic and administrative information
DRUG: drug Information
FARES: FDA Adverse Event Reporting System
FDA: Food and Drug Administration
GnRH: gonadotropin-releasing hormone
MedDRA: Medical Dictionary for Regulatory Activities
MGPS: multi-item gamma Poisson shrinker
PRR: proportional reporting ratio
PS: primary suspect drug
PS: primary suspected drug
PTs: preferred terms
REAC: preferred terminology for adverse drug reactions
ROR: reporting odds ratios
SOC: System Organ Class
